# Potentials of curcumin against polycystic ovary syndrome: Pharmacological insights and therapeutic promises

**DOI:** 10.1016/j.heliyon.2023.e16957

**Published:** 2023-06-02

**Authors:** Tanzina Akter, Md. Sarwar Zahan, Nafisa Nawal, Md. Hasanur Rahman, Tayyabatun Nur Tanjum, Kazi Ifthi Arafat, Akhi Moni, Mohammad Nazrul Islam, Md Jamal Uddin

**Affiliations:** a ABEx Bio-Research Center, East Azampur, Dhaka-1230, Bangladesh; b Department of Biotechnology, Sher-e-Bangla Agricultural University, Sher-e-Bangla Nagar, Dhaka-1207, Bangladesh

**Keywords:** PCOS, Hyperandrogenism, Oxidative stress, Insulin resistance, Inflammation, Hyperglycemia, Hyperlipidemia, Apoptosis

## Abstract

Polycystic ovary syndrome (PCOS) is a common hormonal disorder among women (4%–20%) when the ovaries create abnormally high levels of androgens, the male sex hormones that are typically present in women in trace amounts. The primary characteristics of PCOS include oxidative stress, inflammation, hyperglycemia, hyperlipidemia, hyperandrogenism, and insulin resistance. Generally, metformin, spironolactone, eflornithine and oral contraceptives are used to treat PCOS, despite their several side effects. Therefore, finding a potential candidate for treating PCOS is necessary. Curcumin is a major active natural polyphenolic compound derived from turmeric (*Curcuma longa*). A substantial number of studies have shown that curcumin has anti-inflammatory, anti-oxidative stress, antibacterial, and anti-apoptotic activities. In addition, curcumin reduces hyperglycemia, hyperlipidemia, hyperandrogenism, and insulin resistance in various conditions, including PCOS. The review highlighted the therapeutic aspects of curcumin against the pathophysiology of PCOS. We also offer a hypothesis to improve the development of medicines based on curcumin against PCOS.

## 1 Introduction

Polycystic ovary syndrome (PCOS) is a complex, well-recognized, reproductive, and heterogeneous endocrine disorder that affects approximately 8–20% of child-bearing aged women worldwide [1,2]. The prevalence of PCOS increased by 4.47% within a decade (2007–2017) [3]. The prevalence of PCOS in the southern United States was 47.5% [4]. South Asian women had the highest prevalence (52%) compared to the other regions [5].

PCOS is a phenotype characterized by a self-reinforcing vicious cycle of neuroendocrine, metabolic, and ovarian dysfunction [6]. The pathophysiology of PCOS includes genetic and epigenetic changes, ovarian abnormalities, and neuroendocrine alterations. Primary abnormalities in the hypothalamic-pituitary axis, insulin secretion, and ovarian function are involved in the pathophysiology of PCOS [7,8]. In addition, PCOS is linked to hormonal imbalances such as hyperandrogenemia (HG), insulin resistance (IR), and hyperinsulinemia [7,9]. 60–95% of women with PCOS have insulin resistance, aggravated by increased visceral adiposity [10]. The visceral adipocytes secrete several molecules, including inflammatory markers, resulting in low-grade inflammation [11]. In PCOS, the ovaries produce up to 60% of the androgens, while the adrenals contribute to the remaining 40% [12]. The disrupted gonadotrophin-releasing hormone (GnRH) secretion pattern increases luteinizing hormone (LH) compared to follicle-stimulating hormone (FSH) [13,14]. Lower FSH levels inhibit follicular maturation and thus ovulation, whereas increased LH pulse frequency enhances theca cell androgen synthesis and is involved in cyst formation [15,16]. Elevated androgen results in hyperandrogenemia, hirsutism, anovulation (40% of women), infertility, and polycystic ovaries or hyperthecosis [17,18]. Further, PCOS is linked to oxidative stress, characterized by the production of free radicals and a decrease in serum total antioxidant levels [19].

Medical management focuses on an integrative approach because pharmaceutical treatments demonstrate moderate effectiveness in symptomatic treatment [[20], [21], [22], [23], [24]]. Nowadays, oral contraceptive pills are the most frequently used medication for PCOS. They diminish the amount of free androgen in blood circulation and inhibit gonadotropin secretion. In the recent decade, women's use of complementary medicine has escalated, ranging between 26% and 91% [[25], [26], [27], [28]]. Herbal medicine is one type of well-known complementary medicine [28,29]. By improving ovarian function and irregular menstrual cycle, the herbal medicines *Trigonella foenum-graecum* L.*, and Grifolafrondosa* elevate ovulation and fertility [30]. Ingestion of *Punica granatum* L. and *Camellia sinensis* L. juice may lessen body mass index (BMI), serum insulin level, and insulin resistance [31]. However, a small number of PCOS patients who took *Grifolafrondosa* encountered mild pain and distention in the epigastric region [30]. A report stated the side effects of berberine include mild gastrointestinal discomfort, nausea, and constipation [32]. Furthermore, some herbal medicines that alleviate PCOS symptoms may have adverse effects. However, currently, there is no effective medication for treating PCOS.

Curcumin is a yellow polyphenol. It is extracted from the rhizome of a tropical Southeast Asian plant named turmeric [33]. Some recent experiments showed that curcumin is an anti-inflammatory, anti-diabetic and anti-obesity agent in obese and diabetic mouse models [34]. Curcumin has beneficial effects on various female reproductive disorders such as PCOS, ovarian diseases and endometriosis [35]. Mohammadi et al. showed that the anti-inflammatory and antioxidant benefits of curcumin on PCOS may be attributed to its inhibitory influence on tumor necrosis factor-alpha (TNF-α), serum interleukin-6 (IL-6), and *C*-reactive protein (CRP) expression levels [36]. In another study, Sohaei et al. found that curcumin supplement improved serum insulin and quantitative insulin sensitivity check index (QUICKI) in a clinical trial conducted on 60 women [37]. Another study reported that curcumin therapy for women with PCOS for 12 weeks improved body weight, glycemic management, blood lipids except for triglycerides and very low-density lipoprotein (VLDL)-cholesterol levels, Peroxisome proliferated-activator receptor gamma (PPAR-γ) and low-density lipoprotein receptor (LDLR) gene expression [38]. Curcumin even reduced oxidative stress and apoptosis-related complications in patients with PCOS [39]. Recent studies reviewed the effect of curcumin on glycemic control and lipid profiles in PCOS [[40], [41], [42]]. However, in this study, we reviewed the data on the protective effects and intrinsic mechanisms of curcumin against the pathophysiology of PCOS.

## 2 Methods

**Fig. 1:**
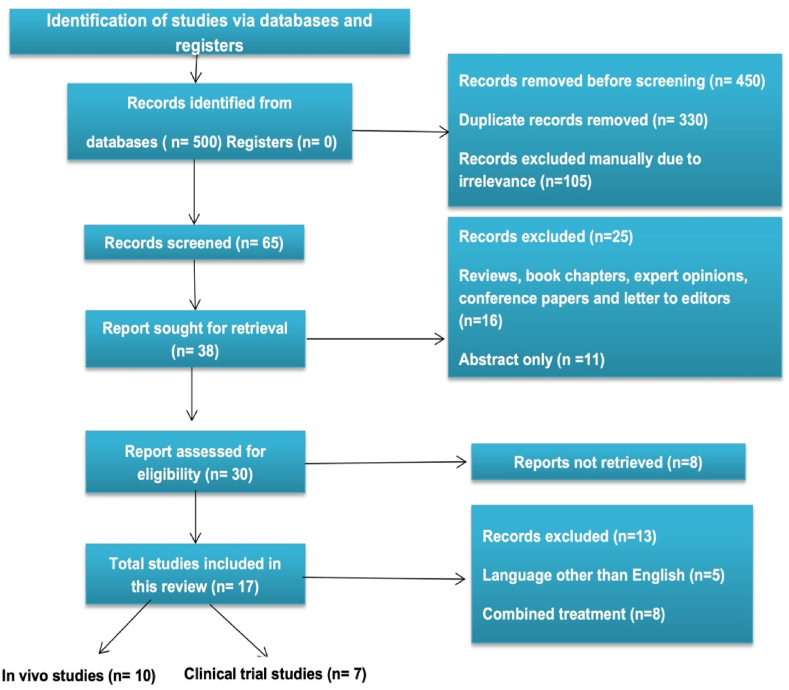
Methods of the reviewing using PRISMA 2020 flow diagram.

This systematic review was carried out following the Preferred Reporting Items for Systematic Reviews and Meta-Analyses (PRISMA) guidelines (Fig. 1) [43]. The literature was collected from published online research databases such as Scopus, PubMed, and Google Scholar using the keywords ‘curcumin on PCOS’ and ‘curcumin on oxidative stress, inflammation, lipid state, apoptosis, hyperglycemia, and hyper androgen etc. The information was retrieved from 2016 to Jan 15, 2022. Some of the items were eliminated using automatic search tools, while others were personally reviewed. Non-English language publications of articles were not included. This evaluation did not include any reviews, book chapters, expert comments, conference papers, or letters to the editors. All figures were generated using MS Power Point.

## 3 Pharmacological effects of curcumin ON PCOS

**Fig. 2:**
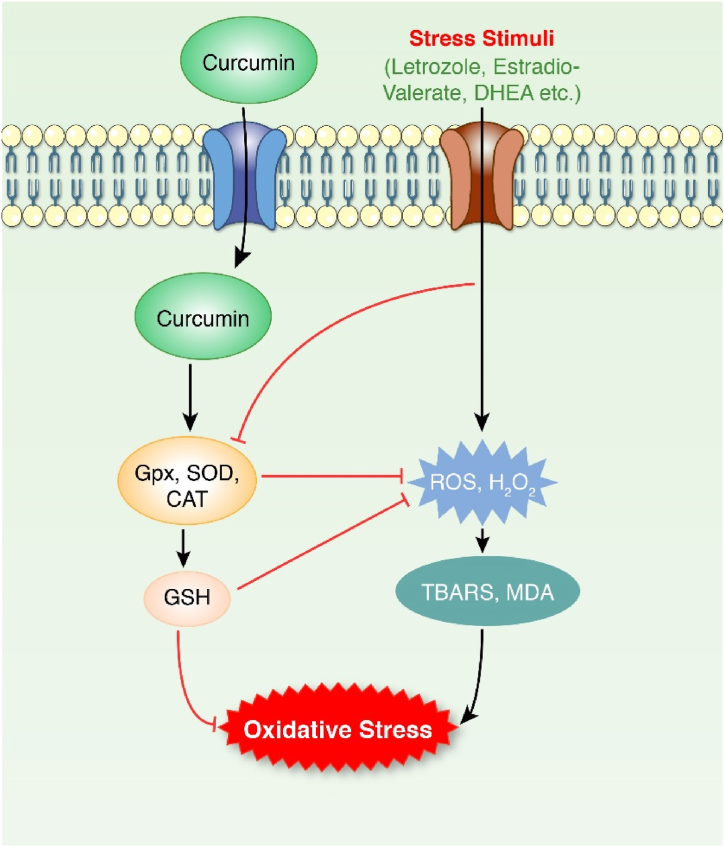
Anti-oxidative effect of curcumin. Stress stimuli (Letrozole, estradiol-valerate and DHEA) activated MDA, TBARS and glutathione via triggering ROS, H_2_O_2_. Oxidative stress emerged as a result of these events. On the other hand, in curcumin-induced models, the expression of CAT, SOD, and GPX are activated, which then activate GSH. These stressors inhibit the expression of oxidative stress suppressive factors. ROS, and H_2_O_2,_ related to oxidative stress, were decreased by GSH. GSH is also capable of reducing oxidative stress.

**Fig. 3:**
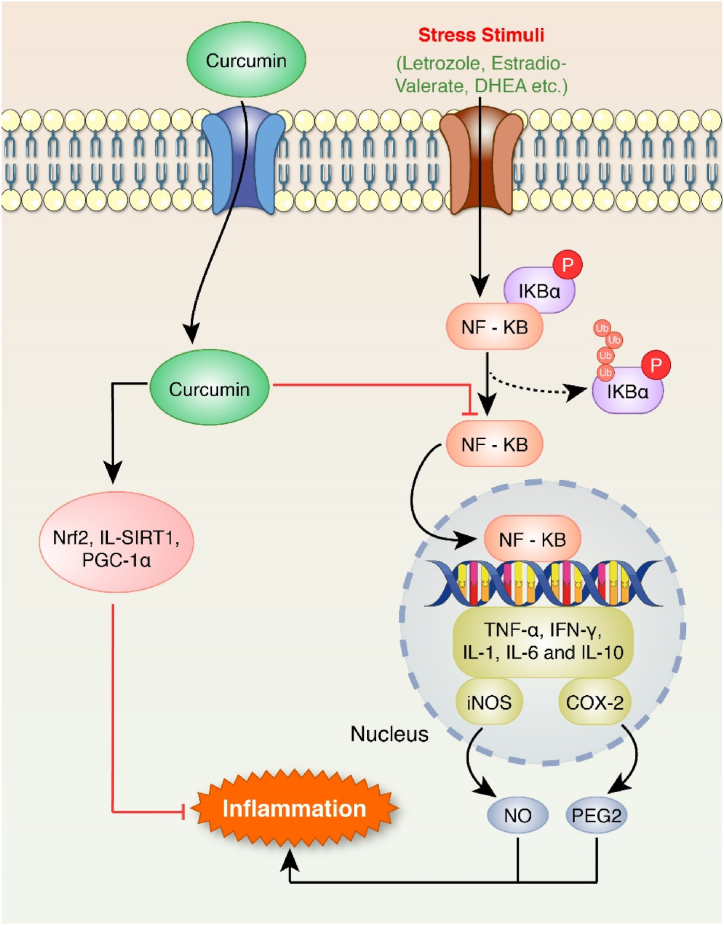
Anti-inflammatory effect of curcumin. The stress stimuli increased NF-кB binding activity. NF-кB in cytosols goes to the nucleus, binds to DNA, and activates TNF-alpha, IFN-γ, IL1, IL-6, IL-10 and COX-2. iNOS is also expressed, which next activates NO. These markers are associated with inflammation. On the other hand, in the curcumin-induced model, Nrf2, IL-SIRT1, and PGC-1 alpha are activated, downregulating the inflammation state. They also downregulate the expression of NF-кB.

**Fig. 4:**
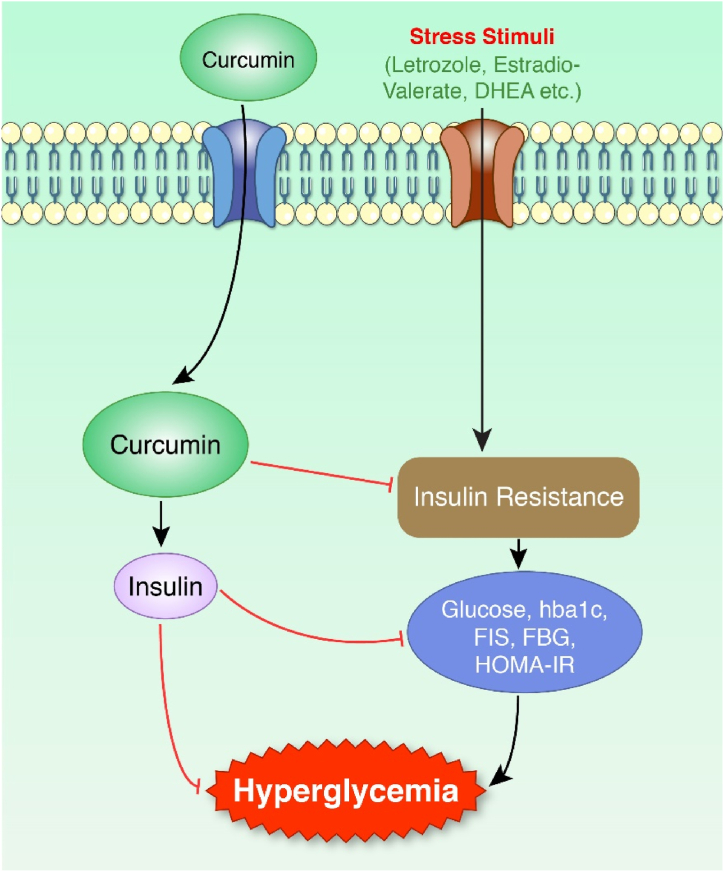
Effect of curcumin against hyperglycemia. Stress stimuli increase insulin resistance via triggering the glucose, HBA1c, FIS, FBG, and HOMA-IR and downregulate insulin production. The Hyper-glycaemia stage is created. On the other hand, insulin production is increased in curcumin-induced models, and the hyperglycemia stage is downregulated.

**Fig. 5:**
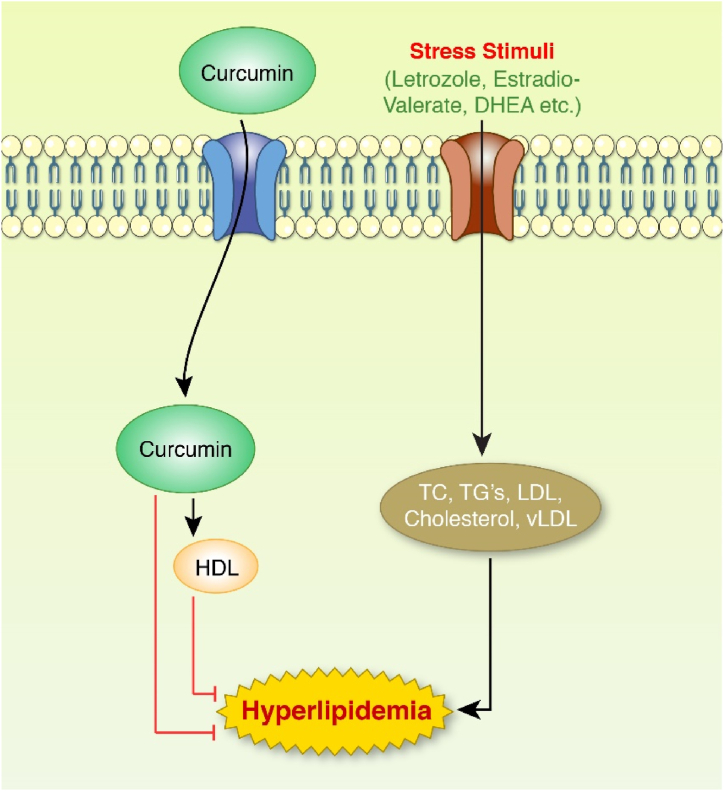
Effect of curcumin against hyperlipidemia. Stress stimuli trigger TC, TG's, LDL, VLDL, and cholesterol. These lipids are available in PCOS patients as they are harmful to health. These stress stimuli inhibit HDL production. On the other hand, they are upregulated by the curcumin-induced model. Curcumin and HDL downregulate hyperlipidemia stage in curcumin-induced models and patients.

**Fig. 6:**
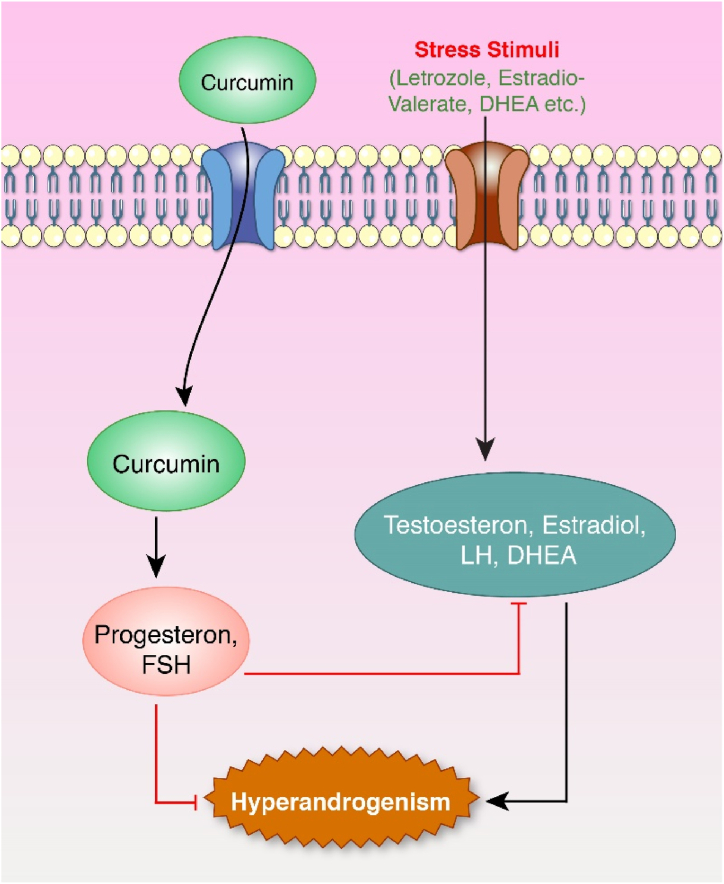
Effect of curcumin against hyperandrogenism. LH, estradiol stimulated by stress stimuli. These hormones stimulate- + androgen testosterone and DHEA, which leads to the hyperandrogenism stage involved in inducing PCOS. In contrast, the curcumin-induced model stimulates progesterone and FSH hormone production. Progesterone lowers the androgen level. FSH increases the binding activity of androgen. Progesterone, FSH together helps to minimize the hyperandrogenism stage.

**Fig. 7:**
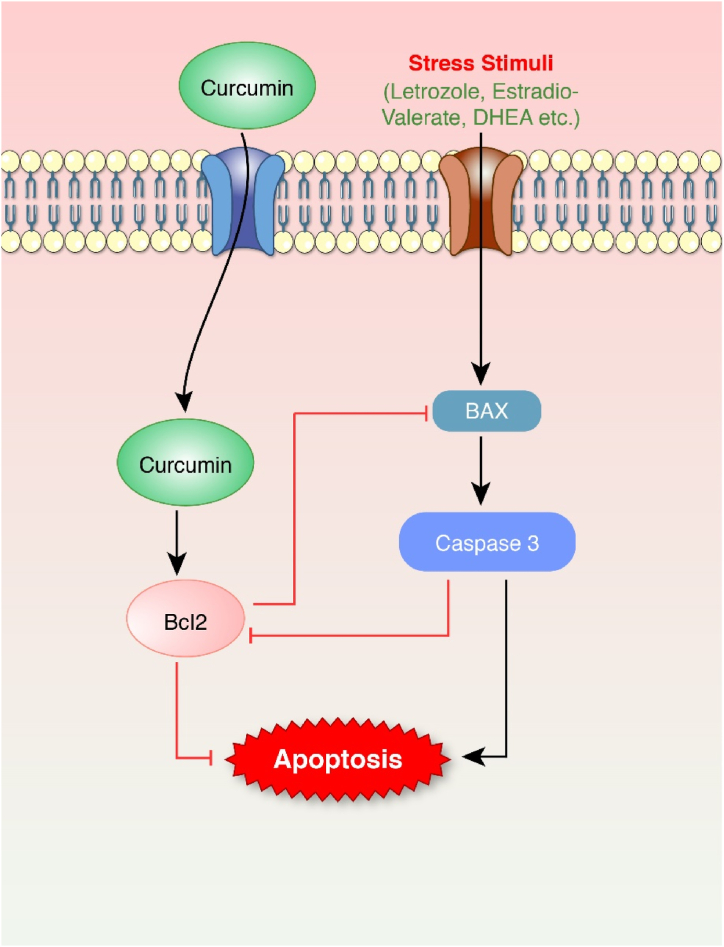
Anti-apoptotic effect of curcumin. Stress stimuli induced BAX and CASP3 production. BAX can transmit cell death via apoptosis through the mitochondrial external membrane. Caspase-3 coordinates cellular structure breakdown, like DNA fragmentation or cytoskeletal protein degradation. In contrast, curcumin stimulated Bcl2 which prevents BAX/BAK oligomerization, thus preventing apoptosis.

The pharmacological potential of curcumin against some plausible factors (such as oxidative stress, inflammation, and other pathologies, as shown in Fig. 2, Fig. 3, Fig. 4, Fig. 5, Fig. 6, Fig. 7 and Table 1, Table 2 responsible for PCOS are summarized in this section.

**Table 1:** Protective effects of curcumin against PCOS in experimental models.

Experimental models	Dose of curcumin	Major research outcomes	Molecular markers	Ref.
Letrozole administered Wistar rats	100 mg/kg and 200 mg/kg BW for 15 days	- Attenuated Letrazole-induced PCOS - Reduced oxidative stress, lipid profile, glucose, and glycosylated hemoglobin levels	↑SOD, ↑catalase, ↑GSH, ↓TBARS, ↓testosterone, ↓estradiol, ↑progesterone, ↓glucose, ↓HBA1c, ↓TC, ↓TG, ↓LDL, ↑HDL	[50]
Estradiol-valerate injected Wistar rats	100 and 300 mg/kg BW for 14 days	- Reduced hepatic inflammation and necrosis - Protected from the inflammatory state of PCOS	↑CRP, ↓TNF-α, ↓COX-2, ↓iNOS, ↓IFN-γ, ↓NF-кB, ↓glucose, ↓insulin, ↓ROS	[36]
Estradiol-valerate injected Wistar rats	600 mg/kg for 14 days	- Reduced cyst and modified hormonal level in PCOS - Exhibited antioxidant and anti-inflammatory properties	↓IL1, ↓ IL-6, ↓ IL-10, ↓TNF-α, ↓NF– KB, ↓NO, ↓iNOS, ↓ COX-2, ↑FSH, ↑progesterone, ↓LH, ↓testosterone, ↓estradiol, ↓triglyceride, ↓cholesterol, ↓LDL, ↓HDL, ↓VLDL	[106]
DHEA administered Sprague–Dawley rats	100 and 200 mg/kg, daily for 30 days	- Alleviated adverse effects of PCOS - Prevented diabetes and decreased insulin resistance	↑GLUT4, ↑Erα, ↓FIS, ↓FBG, ↓HOMA-IR	[71]
Estradiol-valerate injected Wistar rats	100, 200, 300 and 400 mg/kg for 14 days	- Showed anti-inflammatory and antioxidant effects on PCOS - Inhibited TNF-α, serum IL-6 and CRP expression	↑FSH, ↑progesterone, ↓LH, ↓estradiol, ↓testosterone, ↓IL-6, ↓TNF-α, ↓CRP	[134]
Sodium arsenite injected Kunming mice	100,150,200 mg/kg curcumin once per day for 21 days	- May alleviate ovarian oxidative damage - Promoted the proliferation of granular cells	↓ROS, ↓MDA ↑SOD, ↑GPX	[54]
Letrozole administered Wistar rats	100 and 200 mg/kg for 14 days	- Maintained hormonal balance - Restored the ovarian morphology	↑FSH, ↓LH	[114]
DHEA administered mice	5.4 mg/100 g for twenty consecutive days	- Suppressed ovarian injury and DHEA-induced apoptosis	↓BAX, ↓CASP3, ↑Bcl2 ↓insulin	[117]
Letrozole administered Wistar rats	Ingestion for 15 days at doses of 50 mg/kg, 100 mg/kg, and 200 mg/kg	- Improved insulin sensitivity and β-cell integrity - Potential to protect against PCOS pancreatic deficits	↓LDL, ↑HDL, ↓TG ↓insulin ↓MDA, ↑GSH, ↑SOD ↓TNF-α	[51]
Stradiol valerate- administered Wistar rats	300 mg/kg BW for 14 days	- Attenuated inflammatory features of PCOS in liver	↓LDL, ↑HDL	[135]
Letrozole administered adult Wistar rats	Nanocurcumin (200 mg/kg)	- Attenuated inflammation, autophagy activity, and insulin resistance	↓insulin resistance, ↓autophagy activity, ↓NF- kB	[79]

BAX, BCL2 Associated X; Bcl2, B-cell Lymphoma 2; CASP3, Caspase 3; COX-2, Cyclooxygenase isoenzymes; CRP, *C*-Reactive Protein; DHEAS, Dehydroepiandrosterone Sulfate; FBG, Fasting Blood Glucose; FSH, Follicular Stimulating Hormone; Gpx, Glutathione peroxidase; GSH, Glutathione; HBA1c, Hemoglobin A1c; HDL, High-Density lipoproteins; Homeostatic Model Assessment for Insulin Resistance; IL-1, Interleukin-1; IL-6, Interleukin 6; IL-10, Interleukin-10; iNOS, Inducible nitric oxide synthase; LDL, Low-Density Lipoproteins; LH, Luteinizing Hormone; MDA, Malondialdehyde; NF– KB, Nuclear Factor kappa-light-chain-enhancer of activated B cells; NO, Nitric oxide; NPS, nanoparticles; Nrf2, Nuclear Factor Erythroid-2; PGC-1α, Peroxisome Proliferator-activated receptor Gamma Coactivator 1-alpha; ROS, Reactive Oxygen Species; SIRT1, Sirtuin-1; SOD, Superoxide dismutase; SPIONS, Super-paramagnetic iron oxide (Fe3O4); TBARS, Thiobarbituric acid reactive substances; TG, Triglycerides; TNF-α, Tumor Necrosis Factor Alpha; VLDL, Very Low Density Lipoprotein.

**Table 2:** Protective effects of curcumin against PCOS under clinical trial.

Clinical trial	Dose of curcumin	Major research outcomes	Molecular markers	Ref.
Women with PCOS	500 mg–1500 mg per day for 6–12 weeks	- Improve glycemic control and lipid metabolism - Alleviated metabolic abnormality	↓NF-κB, ↓TNF-α, ↓IL-6, ↓DHEA,↑GPx, and ↓insulin,	[40]
Women (18–49 years) with PCOS for at least 2 years	1500‐mg/day for 12 weeks	- Ameliorated PCOS-associated hyperandrogenemia and hyperglycemia	↓FPG, ↓FBS, ↓insulin, ↓LDL, ↑HDL, ↑Estradiol, ↓DHEA	[39]
60 overweighted Women with PCOS	(500 mg twice daily) per day for 6 weeks (orally)	- Improved serum insulin and QUICKI	↓CRP, ↓HOMA-IR, ↓HBA1c, ↓FBS, ↓LDL, ↓serum TG, ↑HDL	[37]
72 overweight, obese PCOS female patients with impaired glucose intolerance	1500 mg/day (500 mg 3 times daily) for 12 weeks	- Reduced oxidative stress-related complications in PCOS	↑GPx, ↑SOD, ↑PGC1α, ↑SIRT1	[55]
30 women who were newly diagnosed with PCOS	Daily dose of 93.34 mg (2 capsules) for 8 weeks	- Improved the anthropometric measurements and glycemic parameters	↓FBG, ↓HOMA-IR, ↓CRP, ↓FSH, ↓LH, ↓Testosterone, ↓DHEAS	[84]
100 women Metformin-induced women (50)	80 mg/day capsule three times daily for 12 weeks	- Alleviated insulin resistance and lipid profile	↓LDL, ↑HDL, ↓TG, ↓ fasting insulin, ↓HOMA-IR, ↓testosterone	[125]
60 women with PCOS aged from 18 to 40 years old	500 mg/day curcumin	- Reduced insulin resistance - Affected body weight and glycemic control	↓LDL, ↑HDL, ↓FPG, ↓HOMA-IR	[38]

BAX, BCL2 Associated X; Bcl2, B-cell Lymphoma 2; CASP3, Caspase 3; COX-2, Cyclooxygenase isoenzymes; CRP, *C*-Reactive Protein; DHEAS, Dehydroepiandrosterone Sulfate; FBG, Fasting Blood Glucose; FSH, Follicular Stimulating Hormone; Gpx, Glutathione peroxidase; GSH, Glutathione; HBA1c, Hemoglobin A1c; HDL, High-Density lipoproteins; Homeostatic Model Assessment for Insulin Resistance; IL-1, Interleukin-1; IL-6, Interleukin 6; IL-10, Interleukin-10; iNOS, Inducible nitric oxide synthase; LDL, Low-Density Lipoproteins; LH, Luteinizing Hormone; MDA, Malondialdehyde; NF-κB, Nuclear Factor kappa-light-chain-enhancer of activated B cells; NO, Nitric oxide; NPS, nanoparticles; Nrf2, Nuclear Factor Erythroid-2; PGC-1α, Peroxisome Proliferator-activated receptor Gamma Coactivator 1-alpha; QUICKI, Quantitative Insulin Sensitivity Check Index; ROS, Reactive Oxygen Species; SIRT1, Sirtuin-1; SOD, Superoxide dismutase; SPIONS, Super-paramagnetic iron oxide (Fe3O4); TBARS, Thiobarbituric acid reactive substances; TG, Triglycerides; TNF-α, Tumor Necrosis Factor Alpha; VLDL, Very Low Density Lipoprotein.

### 3.1 Oxidative stress

During oxidative stress, the intracellular defense reactions are diminished, thus failing to safeguard cells against reactive oxygen species (ROS) (hydroxyl radicals, superoxide anion, hydrogen peroxide, etc.). These events eventually lead to irreparable cellular injury. ROS, the highly reactive derivatives of molecular oxygen, is formed by successive reductions of oxygen [44]. ROS synthesis and breakdown occur in the cells in a balanced manner. Disruption in this balance can accelerate the oxidative stress state in cells [[45], [46], [47]].

Different studies have reported curcumin as a potent antioxidant agent and free radical scavenger (Fig. 2). The functional groups of curcumin that contribute to the antioxidant activity are the hydroxyl (-OH) group and methylene (–CH_2_–) group of the β-diketone moiety [48]. In the estradiol-valerate induced PCOS in Wistar rats, curcumin treatment declined ROS production [36]. Additionally, in a physiological state, several antioxidants protect against the deleterious effect of oxygen-free radicals. Examples of well-known antioxidants include glutathione (GSH), water-insoluble vitamin E, water-soluble vitamin C, and some endogenous enzymes (glutathione-*S*-transferase, superoxide dismutase, catalase, and glutathione peroxidase) [49]. A study on letrozole-treated female Wistar rats showed a high level of oxidative markers and reduced antioxidant enzyme activity such as superoxide dismutase (SOD), GSH, and catalase. After curcumin treatment, the activity of these enzymes was increased [50,51]. Oxidative stress produces free radicles leading to lipid peroxidation by attacking the polyunsaturated fatty acid. Lipid peroxidation produces thiobarbituric acid reactive substances (TBARS) and malondialdehyde (MDA) as by-products. Hence, TBARS and MDA are oxidative stress markers [52,53]. According to a study, TBARS synthesis increased in female model rats with PCOS. The level reached normal with curcumin treatment [50]. Sodium arsenite-induced oxidative stress caused gradual elevation in oxidative stress markers in Kunming mice [54]. Subsequent curcumin treatment reduced ROS synthesis. However, the antioxidant enzyme SOD and Gpx increased in curcumin-treated mice [54]. Besides, an increased MDA level was demonstrated in sodium arsenite and letrozole-induced model animal studies, respectively. But curcumin minimized the MDA level in both cases [51,54].

A clinical trial conducted on female patients with PCOS reported higher Gpx and SOD activity, followed by curcumin therapy [40,55].

### 3.2 Inflammation

Chronic inflammation contributes to the etiology of PCOS. The inflammatory process is caused by the augmentation of several inflammatory elements, such as pro-inflammatory cytokines and chemokines [[56], [57], [58]]. TNF-α is an inflammatory cytokine that is crucial for both ovulation and pregnancy. This cytokine is commonly found in theca cells, macrophages, granulosa cells and oocytes. It triggers the proliferation of follicular theca cells. But, excessive expression of TNF-α in adipose tissue may result in high blood glucose levels and insulin resistance [59]. It can influence the insulin signaling pathway and develop insulin resistance [60,61]. TNF-α causes insulin resistance by stimulating serine phosphorylation of insulin receptor substrate-1(IRS-1) and interfering with the functions of β cells [[62], [63], [64]]. Another cytokine IL-6 regulates inflammation and governs the production of different cytokines [65]. It is essential in ovarian maturation and implantation procedure [59]. The elevated amount of TNF-α followed by excessive production of IL-6 monocytes is found in the serum of women with PCOS, causing low-grade inflammation [66,67].

Curcumin can interact with these cytokines as a potent anti-inflammatory agent (Fig. 3) [68]. It serves as a PI3K/AKT/mTOR signaling pathway inhibitor and, therefore, downregulates the expression of TNF-α along with several pro-inflammatory cytokines [69,70]. The nano-curcumin treatment decreased the serum TNF-α levels in letrozole-induced rat models [51]. Likewise, orally administered curcumin in DHEA-induced Sprague-Dawley female rats significantly reduced pro-inflammatory cytokine production [71]. In another study, curcumin treatment reduced serum levels of CRP, TNF-, and IL-6 in PCOS-induced Wistar rats [72]. Nuclear Factor kappa-light-chain-enhancer of activated B cells (NF-κB) is a ubiquitous and pro-inflammatory transcription factor. It regulates immune response by triggering the synthesis and releasing various cytokines or inflammatory factors, such as TNF-α, IL-6, IL-10, IL-18, TGF-β, and IFN-γ [73]. The amount of NF-кB, TNF- α, COX-2, iNOS kinase and IFN-γ was declined in the PCOS-induced Wistar rat after curcumin treatment. The activation of NF-κB is suppressed by curcumin in different cell lines. This inhibitory effect is produced by restricted I kappa B kinase (IKK) activity and is prompted by TNF, IL-1, hydrogen peroxide (H_2_O_2_), and phorbol ester. Hence, the diminution of NF-кB reduces the activity of certain inflammatory enzymes like iNOS kinase and COX-2 [36]. The activity of peroxisome proliferator-activated receptor gamma coactivator 1-alpha (PGC-1α) is interconnected with inflammation. During the inflammatory state, the activity of PGC-1α is repressed. This event accelerates the inflammatory response [74,75]. Nuclear Factor Erythroid-2 (Nrf2) is a transcriptional factor that inhibits inflammation. It impedes the expression of pro-inflammatory cytokine genes (IL-6 and IL-1b) [76]. SIRT1 inhibits the role of NF-кB complex as a transcription factor as it deacetylates the RelA/p65 (lysin-310 residue) [77]. The overexpression of SIRT1 reduced IL-6 and TNF-α by downregulating NF-кB activity in the liver [78]. Curcumin therapy increased the level of SIRT-1, Nrf-2, and PGC-1α in an experiment conducted on patients with PCOS. Also, nanocurcumin dramatically increased the expression of miR-223–3p and decreased NF-kB to replenish ß cell mass in the pancreas of rat model of PCOS [79].

Another outcome of a clinical trial depicted that circulating TNF-α, IL-6 and other pro-inflammatory cytokine levels diminished in women with PCOS after curcumin therapy [40]. CRP, produced by the human hepatocyte, is considered an indicator or marker of the inflammatory process and is positively regulated by TNFα and IL-6 [80]. This acute-phase reactant acts as an inflammation mediator by inducing endothelial dysfunction and promoting MCP-1-mediated chemotaxis [81]. Moreover, an elevated amount of high sensitivity CRP is a significant prognosticator of cardiovascular disease and is closely associated with insulin resistance [82,83]. After curcumin supplementation therapy, women with PCOS demonstrated decreased CRP expression in two separate studies [37,84].

### 3.3 Hyperglycemia

PCOS is a metabolic disease linked with type 2 diabetes mellitus (DM2) that begins with hyperglycemia and progressively leads to insulin resistance [50,85]. Insulin resistance affects up to 70% of women with PCOS [61]. Women with PCOS develop impaired glucose metabolism at an earlier age and may progress more quickly from impaired glucose tolerance (IGT) to DM2 [86]. Insulin resistance is often accompanied by an increase in endogenous insulin synthesis as a compensatory mechanism. Insulin resistance is linked to high amounts of endogenous insulin, which leads to weight gain, eventually exacerbating insulin resistance [87,88]. This vicious cycle continues until pancreatic beta-cell activity can no longer match the increased insulin demand caused by insulin resistance, resulting in hyperglycemia. Glycemic levels rise to levels compatible with DM2 when the mismatch between insulin demand and production persists, called hyperglycemia. Curcumin has increased insulin sensitivity substantially and reduced insulin resistance (Fig. 4) [89]. In addition, glucose tolerance is promoted by curcumin treatment in certain experimental models [90]. According to direct experimental data, curcumin increases glucose tolerance by stimulating glucagon-like peptide-1 (GLP-1) production [91]. Curcumin has anti-inflammatory and antioxidant properties, which are vital in improving beta-cell functions [92]. Beta cell secretes insulin which assists glucose uptake by the liver or muscle from blood [93]. Therefore, blood glucose level remains balanced.

Moreover, curcumin shows antidiabetic effects in the liver by increasing glycolysis and glycogen synthesis while decreasing gluconeogenesis and in the skeletal muscle by increasing glucose absorption, glycolysis, and glycogen synthesis [94]. Curcumin can lower blood glucose levels by reducing endogenous glucose production, suppressing hyperglycemia-induced inflammation, and stimulating glucose uptake. Additionally, curcumin reduces blood glucose by upregulating the expression of the glucose transporter type 4 (GLUT4), GLUT2, and GLUT3 genes, activating AMP kinase, promoting peroxisome proliferator-activated receptor (PPAR) ligand-binding activity, stimulating insulin secretion from pancreatic tissues, improving pancreatic cell function, and lowering insulin resistance [95]. The glycemic profile of the curcumin-treated experimental model is reported in Table 1. Mice administered with DHEA were treated with 5.4 mg/100 g for twenty consecutive days showed decreased insulin levels [96]. Curcumin treatment reduced fasting blood glucose (FBG), fasting insulin (FINS), and homeostatic model assessment of insulin resistance (HOMA-IR) in DHEA-treated Sprague-Dawley female rats [71]. Female Albino Wistar rats were treated with curcumin for 15 days and decreased letrozole-induced glucose and hb1c level [50,51]. In another study, curcumin treatment reduced blood glucose levels and insulin in estradiol-valerate injected Wistar rats [36]. Nanocurcumin also attenuated the insulin resistance in letrozole-induced Wistar rats [79]. A group of PCOS-affected women, varying from 18 to 40 years old, were treated with curcumin for 12 weeks, which decreased fasting plasma glucose (FPG), HOMA-IR and increased QUICKI [38]. Women with PCOS aged 18 to 49 were treated with curcumin for 12 weeks and manifested a low level of FPG, FBS and insulin [39]. In another clinical trial of 60 women with PCOS, curcumin treatment reduced HOMA-IR, FBS and HbA1c [37].

### 3.4 Hyperlipidemia

Women suffering from PCOS may possess lipid abnormalities. According to a recent study, women with PCOS typically have moderate hypercholesterolemia [97]. PCOS has different lipid patterns, including low levels of high-density lipoprotein cholesterol (HDL-C), high triglyceride (TG), total cholesterol (TC), and low-density lipoprotein cholesterol (LDL-C), as well as considerably greater lipoprotein concentrations [98,99]. The C677T polymorphism of the methylenetetrahydrofolate reductase (MTHFR) gene reduces MTHFR enzyme activity, leading to hyperhomocysteinemia, which is linked to hyperlipidemia [100].

Curcumin exerts cholesterol-lowering effects on humans and animals. It inhibits the accretion of blood cholesterol concentrations in animal experiments by reducing dietary cholesterol absorption (Fig. 5) [101]. Curcumin mainly lowers blood and hepatic cholesterol levels by blocking the 3-hydroxy-3-methyl-glutaryl-coenzyme a reductase (HMG-CoA reductase) enzyme [102]. Curcumin has been shown to promote CYP7A1 enzymatic activity by raising its hepatic gene expression, resulting in increased cholesterol clearance as bile acids [103]. Curcumin suppressed glycerol release while improving glucose absorption by activating PPARγ and CCAT/enhancer-binding protein-α (CEBPA) [104]. A recent study reported that curcumin inhibited the production of *ldlr* (the gene that encodes for the protein known as the low-density lipoprotein receptor) and decreased the absorption of extracellular LDL via inhibiting sterol regulatory element-binding protein-2 (SREBP-2) gene expression and activity [105]. The lipid profile of the curcumin-treated experimental model is presented in Table 1. Letrozole-injected female Wister rats were treated with curcumin which decreased TC, TG, LDL and raised the level of HDL [50]. Raoofi et al. demonstrated that triglyceride, cholesterol, LDL, HDL and VLDL were significantly increased in curcumin-treated female Wistar rats [106].

Various studies among PCOS-affected women showed that curcumin lowered TG, LDL and cholesterol in the blood and increased HDL and VHDL [[37], [38], [39],^51^]. Different bioactive components of curcumin have been linked to anti-lipid and other metabolic benefits in animal studies. Tetrahydrocurcumin, ferulic acid and vanillic acid are examples of these, all of which are curcumin metabolites. Curminoids (diarylheptanoid, demethoxycurcumin, and curcuminoids), bisdemethoxycurcumin, desmethoxycurcumin, and methoxycurcumin are different types of curcumin [107,108]. However, the effects of curcumin on HDL, LDL, and triglycerides are still being studied, and further research is needed to confirm these findings [40].

### 3.5 Hyperandrogenism

Hyperandrogenism refers to a condition in which the number of androgens (male hormones) in females exceeds the normal range. Ovarian and extra-ovarian hyperandrogenism are prominent symptoms of PCOS [109]. PCOS is the most prevalent endocrine condition in women of reproductive age, with a frequency ranging from 5% to 15% [110,111]. The hyperandrogenic state in the ovary is caused by androgen production in the ovarian theca cells [109]. Increased ovarian androgen levels are a common sign of hyperandrogenism in PCOS, resulting in poor follicular maturation. Elevated androgens levels may deleteriously influence follicular growth, resulting in atresia. Generally, the ovaries are the primary source of androgen abundance in PCOS patients. However, 20–30% of PCOS individuals have elevated adrenal androgen levels [112,113].

The majority of available PCOS drugs are androgen inhibitors. Several derivatives of curcumin suppress androgen action (Fig. 6). In letrozole-injected female Wistar rats, curcumin inhibited LH and stimulated FSH [114]. In another experimental model of estradiol valerate injected Wister rats, curcumin downregulated LH, testosterone, and estradiol production and upregulated FSH synthesis [106].

Clinical trials of curcumin extract or compounds on women with PCOS exhibited outcomes analogous to animal experiments. In a clinical trial on women with PCOS, curcumin extract or compounds inhibited insulin and dehydroepiandrosterone (DHEA) [40]. Another trial showed that women (18–49 years old) with PCOS for at least two years, curcumin (1500 mg/day for 12 weeks) suppressed DHEA and stimulated estradiol instead of repression [39]. Thirty newly diagnosed women with PCOS were given a daily dose of 93.34 mg (2 capsules) for eight weeks, demonstrating similar inhibition activity by reducing testosterone, LH, FSH, and DHEA [84].

### 3.6 Other pathological effects related to PCOS

According to clinical research, apoptosis is seldom identified in the glandular epithelium during the proliferative phase or at the starting point of the secretory phase of the menstrual cycle [115]. Despite the absence of quantification, the number of apoptotic bodies in epithelial cells in PCOS patients without hyperplasia is higher than in non-PCOS controls and PCOS patients with hyperplasia [116].

In line with this, the following curcumin extracts or isolated compounds have been reported to protect PCOS (Fig. 7). Curcumin compounds have been shown to prevent apoptosis in several investigations. In a study of DHEA administered prepubertal BALB/c mice, curcumin inhibited the activity of BCL2-associated X (BAX), Caspase 3 (CASP3), and insulin but enhanced B-cell lymphoma 2 (Bcl2) expressions [117]. Excess androgen can cause cell death via various signaling mechanisms through Klotho expression [118]. Hyperandrogenism in PCOS may potentially induce apoptosis in oocytes. Heat shock protein 27 (HSP27), an anti-apoptotic protein in the HSP family, is dramatically downregulated in PCOS oocytes [119]. Nanocurcumin also showed to restore the ß cell mass by erupting autophagy [79].

## 4 Future prospects and limitations

**Fig. 8:**
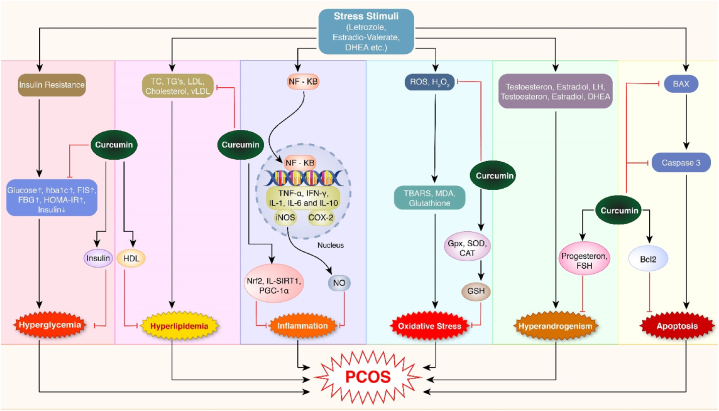
Protective potentials of curcumin against polycystic ovarian syndrome. Letrozole, Estradiol-valerate, DHEA causes PCOS in different pathways (oxidative stress, inflammation, hyperglycemia, hyperlipidemia androgen stress, apoptosis) and those pathways can be inhibited by curcumin. In various research models such as rats, and mice, these stress stimuli increase insulin resistance through increasing insulin resistance factors (HBA1c, glucose, FIS, FBG) which results in hyperglycemia. These stress stimuli also increase LDL, VLDL, TG, etc., which results in hyperlipidemia. The increased rate of NF-ҡB also induce iNOS, NO, COX-2, ILs. These factors indicated inflammation in PCOS. Oxidative stress is another pathway activated via increased ROS production, MDA, etc. Male hormones were increased in response to those stress stimuli, resulting in hyperandrogenism. Another pathway, apoptosis, is stimulated via the increased production of CASP3 and BAX. Curcumin has inhibitory activity against these pathways. Curcumin can regulate the production of insulin resistance factors. It increases the production of HDL, which decreases the condition of hyperlipidemia. Curcumin also increases the anti-inflammatory factors. Curcumin inhibited oxidative stress by upregulating SOD, GPX, GSH, and Catalase. Curcumin can also stimulate the expression of FSH and progesterone, which can control the hyperandrogenism state. Additionally, it increased the expression of the Bcl2 gene, which is an anti-apoptosis factor. Therefore, curcumin can improve the condition of PCOS by regulating the various pathways.

Women with PCOS are susceptible to several metabolic disorders, and the current studies have demonstrated the effectiveness of curcumin against PCOS [120]. Studies on rat models and female patients with PCOS pointed out that curcumin treatment positively influences the PCOS-associated parameters (Fig. 8) [50]. Curcumin treatment reduces the oxidative markers, including ROS, TBARS and MDA, in rat models. It also alters the gene expression of SIRT1 and PGC-1α [36,52,53,55]. In experimental PCOS models, curcumin has enhanced the functionality of different antioxidant enzymes, such as SOD, GSH, catalase and GPX [40,50,51,55]. Curcumin lowers blood glucose, so it can be used in treating diabetes [95]. One study showed that curcumin works synergistically with metformin to improve insulin resistance and lipid profile in PCOS patients. As a result, the combination of metformin and curcumin may have therapeutic value in PCOS patients [125]. A study reviewed that curcumin helps with the treatment of metabolic syndrome, arthritis, anxiety, as well as oxidative and inflammatory diseases [121]. Clinical studies suggest that curcumin use is also quite safe when done continuously for up to 4 months [122,123].

According to previous studies, curcumin did not show any notable adverse effects; instead, it limited the serum cholesterol level [101]. However, some studies did not show any significant effect of curcumin on model rats and female patients [50]. This inadvertent result may due to the low absorption of curcumin [84]. Due to its hydrophobic nature, curcumin has poor absorption and very low solubility in water (0.005%) [84]. Its oral absorption is limited, although its usage with piperine (found in black pepper) enhances its absorption [126]. However, nanocurcumin is more efficient in reducing serum cholesterol due to its *anti*-hyperlipidemic potential [96,124]. Nanocurcumin treatment successfully protected pancreatic tissue from oxidative stress caused by PCOS [51]. Along with anti-cancer potentials, nano-curcumin can be one of the best medications for PCOS treatment. Nanoparticles and lipid/liposome formulations that improve curcumin absorption and bioavailability are being explored as better ways to administer curcumin [127,128]. Recent studies suggest that sonicating curcumin with chitosan, *N*-acetyl histidine, and arginine is a gateway into nanomedicine. It has been more stabilized in watery media than free curcumin. Therefore, this characteristic is attributed to an improved drug delivery system of curcumin-loaded nanoparticles. FSH, LH, and testosterone reached their normal level compared to free curcumin drug-treated or metformin-treated mice models [129].

Considering, the advancement and refining of these technologies will allow for cell-directed curcumin targeting and better treatment outcomes against PCOS.

## 5 Conclusions

PCOS is associated to oxidative stress, inflammation, insulin resistance, and hyperandrogenism, making individuals vulnerable to diabetes, endometrial cancer, and cardiovascular disease [130,131]. The current review illustrates various therapeutic properties of curcumin including anti-diabetic, antioxidant, anti-inflammatory, and anti-androgenic effects (Fig. 8). It plays a remarkable role in alleviating the lipid metabolism and glycemic profile in patients with PCOS and model rats. In addition, curcumin is affordable and readily available [132]. These diversified characteristics of curcumin can enlist it as a promising therapeutic herbal medicine in treating PCOS. However, curcumin has low solubility and poor body pH bioavailability. So, the limitation can be considered an impediment to its widespread use [133]. Bio-compatible nanocurcumin has an increased polarity compared to the natural one [51]. In order to reduce autophagy flare, insulin resistance, and boost ß cell mass, nanocurcumin markedly increased the expression of miR-223–3p in the pancreas of rat model of PCOS [79]. Hence, the formulation of nanocurcumin and additional research for its optimization and application as a therapeutic agent may overcome the hurdle of solubility and bioavailability.

## Author contribution statement

All authors listed have significantly contributed to the development and the writing of this article.

## Data availability statement

No data was used for the research described in the article.

## Declaration of competing interest

The authors declare that they have no known competing financial interests or personal relationships that could have appeared to influence the work reported in this paper.
